# Whole Genome Sequencing of Discordant Monozygotic Twins Reveals Regulatory Variants Associated With Nonsyndromic Orofacial Clefts

**DOI:** 10.1155/humu/2471705

**Published:** 2026-09-23

**Authors:** Emmanuel Temitope Aladenika, Mojisola Olujitan, Tamara Busch, Lord Gowans, Wasiu Adeyemo, Adegbayi Adeola Adekunle, Mekonen Eshete, Oluwafunmi Ajala, Rishitha Gadde, Nina Mba, Azeez Alade, Azeez Butali

**Affiliations:** ^1^ Iowa Institute for Oral Health Research, University of Iowa, Iowa City, Iowa, USA, uiowa.edu; ^2^ Department of Oral Pathology, Radiology and Medicine, College of Dentistry, University of Iowa, Iowa City, Iowa, USA, uiowa.edu; ^3^ Komfo Anokye Teaching Hospital and Kwame Nkrumah University of Science and Technology, Kumasi, Ghana; ^4^ Department of Oral and Maxillofacial Surgery, College of Medicine, University of Lagos, Lagos, Nigeria, unilag.edu.ng; ^5^ Addis Ababa University College of Health Sciences, School of Medicine, Surgical Department, Addis Ababa, Ethiopia, aau.edu.et; ^6^ National Institute of Dental and Craniofacial Research, Bethesda, Maryland, USA

## Abstract

Monozygotic (MZ) twins are expected to share nearly identical genomes, yet a substantial proportion is discordant for complex diseases like nonsyndromic orofacial clefts (nsOFC). This suggests a role for postzygotic discordant genetic variations and other developmental mechanisms. In this study, we performed whole genome sequencing of two MZ twin pairs of African ancestry who were discordant for nsOFC to identify genetic variants that may contribute to phenotypic discordance. Following quality control and genotype‐level filtering, we identified 37,271 variants that were present exclusively in the affected twins. Of these, 431 variants were predicted by CADD to have deleterious effects. Among these, 66 were in protein‐coding regions, including 2 predicted protein‐altering missense variants and splice‐site variants. The remaining 365 variants were in noncoding regions including putative craniofacial enhancers. Some of these variants are rare (MAF < 0.01) and were predicted to alter transcription factor binding or disrupt local chromatin architecture within putative craniofacial enhancers located near genes with established roles in craniofacial development, including *COL27A1*, *MPHOSPH8*, and *SPTBN1*. In addition, some common variants previously associated with noncraniofacial traits were also predicted to affect regulatory activity of putative craniofacial enhancers near craniofacial genes. This raises the possibility of pleiotropic regulatory effects that warrant further investigation. Overall, this study identified a set of candidate coding and regulatory variants in MZ twins discordant for nsOFC,providing targets for future functional investigations. We also proposed an overarching genetic mechanism for the prioritized discordant variants.

## 1. Background

Monozygotic (MZ) twins arise from the division of a single fertilized ovum and are therefore expected to share nearly identical genomic sequences [1]. However, accumulating evidence suggests that early postzygotic events can generate genetic differences between MZ twins that result in somatic variants that are present in one twin but absent in the other [2]. Such variants provide a plausible explanation for phenotypic discordance and may contribute directly to disease etiology [3, 4]. Indeed, approximately 40% of MZ twin pairs are discordant for at least one disease phenotype [5]. Traditionally, twin studies have been used to estimate trait heritability by comparing concordance rates between MZ and dizygotic twins [1]. More recently, the identification of molecular differences between discordant MZ twins has expanded the utility of twin studies beyond heritability estimation to include gene and variant discovery [5].

In discordant twin pairs, the unaffected co‐twin serves as an effective control for shared genetic background and many environmental exposures [1]. This case co‐twin design enables the investigation of nonshared genetic variation that may underlie complex disease phenotypes [2, 5]. One of such complex conditions is orofacial clefts (OFC), which result from defective fusion of facial prominences during embryonic development. Approximately 70% of OFC cases are classified as nonsyndromic orofacial clefts (nsOFC) and exhibit a multifactorial etiology shaped by both genetic and environmental factors [6]. Although sequencing and genome‐wide studies have identified several causal genes and susceptibility loci, much of the genetic architecture underlying nsOFC remains unresolved [7–14]. Molecular analyses of discordant MZ twins have been conducted to identify genetic contributors to clefting. This approach has been successfully modeled in studies of Van der Woude syndrome (VWS), where Kondo et al. identified a pathogenic mutation in interferon regulatory factor 6 (*IRF6*) that was present only in the affected twin of a discordant MZ pair, establishing *IRF6* as a causal gene for VWS [3]. Additional studies of MZ twins with VWS have also reported the complexity of genotype–phenotype relationships. In one of such studies, both twins carried the same *IRF6* missense mutation inherited from their father, yet they exhibited markedly different clinical presentations, ranging from isolated lower lip pits to bilateral cleft lip and palate [15].

Beyond these VWS examples, efforts to identify additional coding mutations underlying discordant OFC cases have been largely unsuccessful [16, 17]. This may indicate that highly penetrant coding variants are relatively rare contributors to OFC risk [5, 18]. Instead, phenotypic discordance may arise from variation in noncoding regulatory regions that influence gene expression without altering protein sequence. These regions are enriched for regulatory elements that act in a tissue‐ and time‐specific manner during development and are called enhancers [19]. Disruption of enhancers has been implicated in multiple developmental disorders in both human and animal models [20, 21].

Enhancers represent a key class of noncoding regulatory elements, controlling the timing, location, and magnitude of gene expression through interactions with transcription factors (TFs) [19]. A subset of enhancers is specifically active during craniofacial development, and these craniofacial enhancers appear to be particularly sensitive to genetic variations [19, 22, 23]. Both rare and common variants within noncoding regulatory regions have been identified in individuals with OFC, supporting their potential contribution to cleft susceptibility [23–26]. One hypothesis for phenotypic discordance in MZ twins is that both individuals share a baseline genetic and environmental risk that is insufficient to cause disease on its own. The presence of additional deleterious variants, whether common, rare (MAF < 0.01), or novel, in either protein‐coding or noncoding regulatory regions in one twin may increase liability beyond a pathogenic threshold.

Therefore, we performed whole‐genome sequencing on two pairs of MZ twins discordant for nsOFC, together with available parental samples from the larger African ancestry cohort. We used the unaffected co‐twin as an internally matched comparator and applied a filtering and prioritization framework to identify candidate discordant variants in coding and noncoding regions. We further evaluated candidate variants using publicly available functional annotation, craniofacial enhancer maps, gene‐expression datasets, and computational predictions of TF binding and chromatin interactions. Given our number of twin pairs, our objective was to identify and prioritize candidate variants rather than establish causality.

## 2. Methods

### 2.1. Sample Procurement

#### 2.1.1. Ethics

Ethical approval was obtained from review boards at the Lagos University Teaching Hospital (ADM/DCST/HREC/VOL.XV/321), Obafemi Awolowo University Teaching Hospital (ERC/2011/12/01), Kwame Nkrumah University of Science and Technology (CHRPE/RC/018/130), and University of Iowa (IRB ID #: 201101720).

#### 2.1.2. Recruitment and Assessment Procedures

Two MZ twin pairs (one Ghanaian and one Nigerian) discordant for nsOFC were recruited as part of a larger study of individuals of African ancestry with nsOFC (Figure S1). Both twin pairs were recruited together with their biological parents, for whom whole‐genome sequencing data were available as part of the larger study. The parental samples were used to assess inheritance patterns of candidate discordant variants. Additional unrelated singleton nsOFC cases and their parents were also available within the larger cohort; however, the present analysis focused specifically on the two discordant MZ twin pairs. The recruitment procedures followed our previous work and the AfriCRAN protocol [10]. Informed consent was obtained from all participants. Each individual underwent detailed clinical assessment, including deep phenotyping and echocardiography to rule out cardiac defects.

### 2.2. Whole‐Genome Sequencing and Quality Control

#### 2.2.1. DNA Collection and Extraction

Saliva samples were collected, and DNA was extracted, quantified, and subjected to standard quality checks before shipment to the Broad Institute for sequencing. Sample handling and processing followed established protocols [8].

#### 2.2.2. Sequencing and Quality Control

Whole‐genome sequencing was performed on the samples of the larger African cohort that included the MZ twins and their parents through the Gabriella Miller Kids First Pediatric Research Consortium at the Broad Institute at an average depth of 30X. Reads were aligned to the GRCh38 reference genome, and variants were called using the Genome Analysis Toolkit (GATK) to identify single nucleotide variants (SNVs) and insertions‐deletions (indels). Variants were stored in VCF files.

Quality control was initially performed at the level of the larger African cohort using PLINK v1.9. Cohort‐level quality control included assessment of genotype missingness, sex concordance, sample identity, and Hardy–Weinberg equilibrium (HWE). HWE was evaluated in the larger cohort. Mendelian consistency was assessed using available parent–offspring relationships in the larger cohort, including the biological parents of the two MZ twin pairs. These analyses were used to identify genotype calls inconsistent with Mendelian transmission.

Following cohort‐level quality control, the two MZ twin pairs and their available biological parents were selected from the larger WGS dataset using VarSeq software for the present analysis.

#### 2.2.3. Confirmation of Zygosity of Twins

We confirmed the zygosity of each twin pair using identity‐by‐descent analysis. Both pairs showed the characteristic IBD pattern of MZ twins, with virtually complete allele sharing across the genome (Table S1).

### 2.3. General Analysis of Discordant Variants

**Figure 1:**
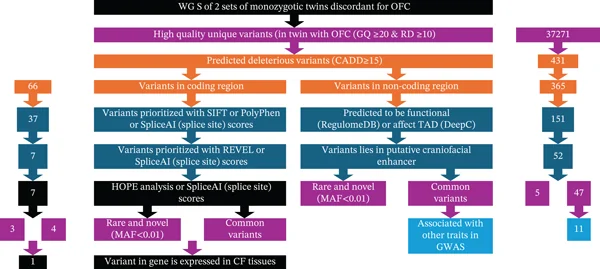
Data filtration pipeline to identify deleterious variants in craniofacial enhancers in two sets of monozygotic twins discordant for OFC. Flanked in the right and left by the number variants at each step.

High‐confidence SNVs were retained using genotype quality ≥ 20 and read depth ≥ 10 (Figure 1). To identify variants potentially responsible for the discordant phenotypes, we compared the variant profiles of each MZ twin pair. For each pair, variants present in the affected twin (Twin 1A or Twin 2A) but absent in the unaffected co‐twin (Twin 1B or Twin 2B) were classified as discordant variants. Variants shared by both twins were excluded, as they were unlikely to explain the phenotypic difference.

The discordant variants were then scored for predicted deleteriousness using the Combined Annotation Dependent Depletion (CADD) score. Discordant variants with CADD ≥ 15 were prioritized, reflecting variants predicted to be among the most damaging in the human genome (https://cadd.gs.washington.edu/) [27] (Figure 1).

### 2.4. Analysis of Prioritized Discordant Variants in the Protein‐Coding Region

#### 2.4.1. Analysis of Discordant Missense Variants

Missense variants were further prioritized using additional in silico prediction tools, including Sorting Intolerant From Tolerant (SIFT; ≤ 0.05) and Polymorphism Phenotyping (PolyPhen; 0.85–1) scores [28, 29]. Variants with a Rare Exome Variant Ensemble Learner (REVEL) score ≥ 0.5 were also given higher priority to increase the likelihood of identifying functionally significant substitutions [30]. To evaluate the potential structural and functional consequences of the pathogenic amino acid changes, we used the Have (y)Our Protein Explained (HOPE) bioinformatics tool (https://www3.cmbi.umcn.nl/hope/) [31]. HOPE analysis simulates the specific mutation and compares the predicted structural and functional impact of the mutant (MT) protein against its wild‐type (WT) counterpart [31]. We interpreted the amino acid changes based on the resulting physiochemical changes in the MT protein and consequent interactions with other molecules [31] (Figure 1).

#### 2.4.2. Analysis of Discordant Splice Site Variants

For splice‐site variants, SpliceAI was used to prioritize because it is among the top‐performing tools for predicting splice‐altering mutations [32–35]. SpliceAI scores were obtained using the SpliceAI webserver from the Broad Institute (https://spliceailookup.broadinstitute.org/) using the options: hg38, masked scores, maximum distance 50 bp. A threshold of > 0.20 was used to prioritize which is consistent with published recommendations that demonstrate that these cutoff balances sensitivity and specificity for identifying splice‐disruptive variants [32] (Figure 1).

#### 2.4.3. MAF Filtering of Variants

Variant allele frequencies were obtained from the Genome Aggregation Database (https://gnomad.broadinstitute.org/), including both the global population frequency and the African/African American population‐specific frequency. Variants were categorized as rare according to predefined frequency thresholds (MAF < 0.01) in the global population.

#### 2.4.4. Investigation of Genes With Prioritized Discordant Variants

We further investigated the genes harboring prioritized discordant variants to determine their relevance to craniofacial development. Gene expression in craniofacial tissues was assessed using the Systems tool for craniofacial expression‐based gene discovery (SysFACE) track of the UCSC genome browser. This SysFACE expression data is based on publicly available databases, including FaceBase (https://www.facebase.org) and the NCBI Gene Expression Omnibus (GEO) (https://www.ncbi.nlm.nih.gov/geo/), that were meta‐analyzed [36] (Figure 1).

We also investigated whether these genes have mouse models with craniofacial phenotypes by mining the Mouse Genome Informatics (MGI) database [37].

### 2.5. Analysis of Prioritized Discordant Variants in the Noncoding Region

#### 2.5.1. Prediction of Functional Impact of Noncoding Discordant Variants

Noncoding variants were first evaluated for their potential to alter TF binding using RegulomeDB. Variants with a RegulomeDB rank score of ≤ 2 were prioritized, as this threshold corresponds to variants with evidence to likely affect TF binding [38]. To assess whether noncoding variants may disrupt local chromatin architecture, particularly through perturbation of CTCF‐mediated boundaries, we used the deep learning–based chromatin interaction model deepC [39]. The model was trained on GM12878 Hi‐C data following the original implementation [39]. For each variant, we performed an in silico substitution of the reference allele with the alternate allele and calculated the average absolute change in predicted chromatin contact scores across the region. We used an average absolute change cut‐off score of 0.01. Visual inspection of the deepC interaction plots was also used to confirm and interpret predicted disruptions in chromatin architecture (Figure 1).

#### 2.5.2. Selection of Putative Craniofacial Enhancers

We used publicly available 18‐state chromatin segmentation datasets generated for multiple Carnegie stages (CSs) (CS13, CS14, CS15, CS17, CS18, CS19, CS20, CS22, and CS23) [40, 41]. For each developmental stage, multiple chromatin segmentation files representing calls from different biological replicates were available. For each stage, we first combined the multiple individual files into a single dataset. Chromosome X coordinates were excluded to restrict the analysis to autosomes. To identify robust enhancer regions supported across replicates, we used bedtools to merge overlapping coordinates across the combined files. Merged coordinates were then filtered to retain only those with at least two overlapping enhancer coordinate calls. The resulting lists represented high‐confidence, stage‐specific putative craniofacial enhancer elements. These putative enhancer datasets were used for all downstream analyses. We then investigated whether any of the prioritized noncoding variants were in these putative craniofacial enhancers.

#### 2.5.3. MAF Filtering of Noncoding Discordant Variants

Variant allele frequencies were obtained from the Genome Aggregation Database (https://gnomad.broadinstitute.org/), including both the global population frequency and the African/African American population‐specific frequency. Variants were categorized as rare according to predefined frequency thresholds (MAF < 0.01) in the global population.

Rare and novel variants were directly included among prioritized variants. Common variants were investigated for genome‐wide significant associations in the GWAS Catalog (http://www.ebi.ac.uk/gwas/), and those linked to any trait were retained for further analysis.

#### 2.5.4. Identification of Craniofacial Genes Near Enhancers With Prioritized Noncoding Discordant Variants

We identified genes located in the same topologically associated domain (TAD) as the craniofacial enhancers with prioritized noncoding discordant variants by mining the UCSC Genome Browser. The relevance of these genes to craniofacial development was assessed using data from MGI [37]. For enhancers located in gene deserts, the nearest genes with known or suspected craniofacial roles were identified.

To predict the target genes of prioritized enhancers, we used the Activity‐by‐Contact (ABC) model, which utilizes chromatin accessibility (ATAC‐seq or DNase‐seq), enhancer activity (H3K27ac), Hi‐C contact frequency data, and RefSeq gene annotations [42]. As a cellular proxy for embryonic oral epithelium, we used publicly available datasets from primary normal human epidermal keratinocytes (NHEK), including H3K27ac (GSM733674), DNase hypersensitivity (GSM736545), and Hi‐C (GSE63525) [43]. ABC scores > 0.02 were considered regulatory interactions between an enhancer and the promoter of a gene, which is consistent with the threshold recommended in the original ABC model publication [42] (Figure 1).

#### 2.5.5. Predicting TFs Affected by Prioritized Noncoding Discordant Variants

To identify TFs whose binding may be altered by variants located within prioritized craniofacial enhancers, we used the JASPAR 2022 database [44] and the TFBSTools package [45]. For each variant, we extracted a 50 bp window centered on the variant and compared predicted transition factors binding sites (TFBS) between the reference and alternate sequences. A TFBS was considered altered if it was statistically significant in only one of the two sequences after correction for multiple testing (*p* < 0.05/number of overlapping TFBS).

We also used the FABIAN‐variant tool to investigate changes in TF binding potential [46]. FABIAN scores the WT and MT sequences independently for potential TF binding changes. Higher WT scores indicate reduced binding, whereas higher MT scores indicate increased binding. FABIAN also provides a joint score from −1 (loss of TFBS) to 1 (gain of TFBS).

#### 2.5.6. Transcriptomics Data Analysis

To assess the coexpression of affected TFs and their predicted target genes, we analyzed publicly available single‐nucleus transcriptomic datasets from human craniofacial tissues at CS17 and CS20, as well as mouse craniofacial tissues at embryonic day E12.5 [40].

### 2.6. Confirmation of Prioritized Coding and Noncoding Discordant Variants

For prioritized discordant variants, read‐level evidence was confirmed and reported at the variant loci using the aligned sequencing data. Total read depth, reference read counts, alternate read counts, and variant allele fractions were reported for both twins (Table S2).

## 3. Results

### 3.1. WGS Data and Initial Variant Prioritization

WGS data from two MZ twin pairs discordant for nsOFC were analyzed as part of a larger African nsOFC cohort. Following cohort‐level quality control, the two MZ twin pairs and their available biological parents were selected for the present analysis. All four twins met the quality‐control criteria for inclusion and were confirmed to have African ancestry by principal component analysis (PCA). Identity‐by‐descent analysis confirmed the MZ status of both twin pairs (Table S1).

Following genotype‐level filtering for high‐confidence variant calls (genotype quality ≥ 20 and read depth ≥ 10), we identified 37,271 discordant variants across the two twin pairs (Figure 1). These variants represented differences in genotype between the affected and unaffected members of each MZ twin pair. To prioritize variants with potential functional effects, variants were subsequently filtered based on a CADD score ≥ 15, resulting in 431 variants predicted to have potentially deleterious effects. These prioritized variants were used for further downstream analyses (Figure 1).

### 3.2. Discordant Variants in the Protein‐Coding Region

Among the 431 prioritized discordant deleterious variants, 66 of them were located within protein‐coding regions (Figure 1 and Table S3). These included 32 missense variants predicted to be deleterious by either SIFT or PolyPhen and five splice‐site variants predicted by SpliceAI to disrupt normal splicing.

Further prioritization of missense variants using REVEL scores identified one variant, chr20:25278370G>T, that exceeded the predefined threshold (REVEL ≥ 0.5). An additional missense variant, chr5:140842949C>A, was also retained for downstream analyses because its REVEL score (0.498) was close to the cutoff (Figure 1 and Table 1).

**Table 1:** List of discordant deleterious variants in coding regions which are predicted to be harmful by other in silico tools (SIFT/PolyPhen, REVEL, and SpliceAI).

Variant	Gene	HGVS c.	HGVS p.	Effect	MAF (Global)	MAF (Africans)	CADD score	SIFT score	PolyPhen Score	SpliceAI score	REVEL score	Twin pair
5:140842949	*PCDHA8*	c.1628C>A	p.Pro543Gln	Missense variant	0.003256	0.060805	20.6	0	—	—	0.498	Twin 1
20:25278370	*PYGB*	c.907G>T	p.Ala303Ser	Missense variant	0.1746	0.076581	32	0	0.815	—	0.762	Twin 2
7:121267212	*CPED1*	—	—	Splice site (acceptor)	0.001662	0.027117	19.18	—	—	0.31	—	Twin 1
7:67278874	*SPDYE21*	—	—	Splice site (donor)	0.092093	0.059184	17.61	—	—	0.96	—	Twin 2
8:33513081	*TTI2*	—	—	Splice site (donor)	0.014697	0.053067	21.9	—	—	0.94	—	Twin 1
17:20501924	*KRT16P3*	—	—	Splice site (donor)	0.005914	0.059718	15.62	—	—	0.78	—	Twin 2
19:28727576	LOC100420587	—	—	Splice site (donor)	0.173455	0.08506	15.29	—	—	0.32	—	Twin 1

**Figure 2:**
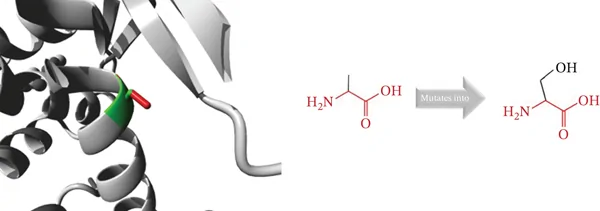
Structural impact of the discordant variant in *PYGB* gene as predicted by HOPE (Have yOur Protein Explained). The wild‐type residue (green side chain) and the variant residue (red side chain) are shown. The variant residue is larger than the wild‐type residue and is predicted to be incompatible with the protein core. In addition, the wild‐type residue is more hydrophobic than the mutant residue.

For the first prioritized missense variant (chr20:25278370G>T) which is in brain‐type glycogen phosphorylase (*PYGB*) (p.Ala303Ser), HOPE predicted that the amino acid substitution from alanine to a larger and less hydrophobic serine could alter local physicochemical properties (Figure 2). Because this residue is buried within the protein core, the change is predicted to disrupt tight core packing and reduce stabilizing hydrophobic interactions, potentially compromising protein stability. Expression analysis using SysFACE shows that *PYGB* is expressed in mouse maxillary and frontonasal tissues at embryonic stages E12 and E12.5 (Figure S2). However, no mouse knockout models for *PYGB* have been reported to exhibit craniofacial abnormalities.

The second prioritized missense variant (chr5:140842949 C>A) in protocadherin alpha 8 (*PCDHA8*) results in a substitution that replaces a rigid proline with a smaller, more flexible glycine, a change predicted to increase local structural flexibility and disrupt proper folding of the domain (Figure S3). The reduction in hydrophobicity may further weaken stabilizing interactions. SysFACE analysis did not show expression of *PCDHA8* in mouse craniofacial tissues, and no mouse models with craniofacial phenotypes have been reported.

The five splice‐site discordant variants were located within 10 kb of exon–intron boundaries of *CPED1*, *PMS2P4*, *TTI2*, *KRT16P3*, and LOC100420587 (Figure 1 and Table 1). These genes were not shown by SysFACE to be expressed in craniofacial tissues and do not have reported craniofacial phenotypes in mouse knockout models.

### 3.3. Discordant Variants in Noncoding Region

**Figure 3:**
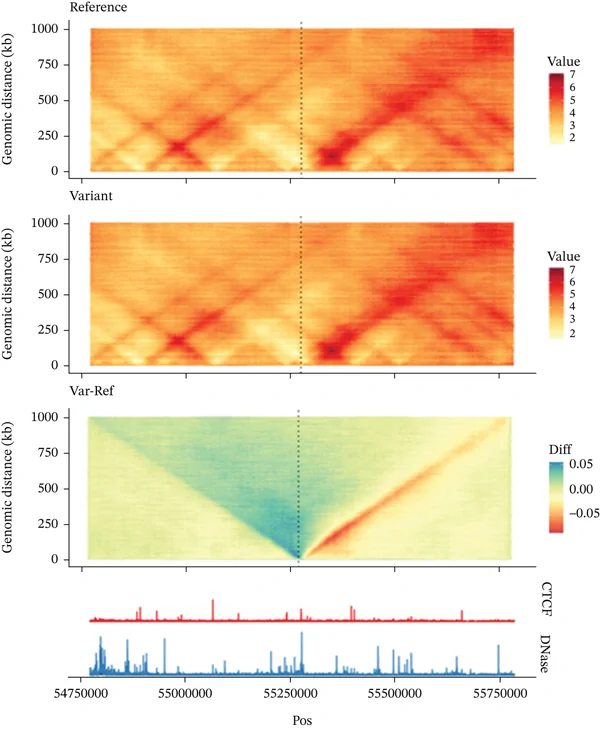
Predicted impact of the variant chr2:55049086 using the deepC model trained in GM12878 cells. Shown are deepC predictions for the reference and alternate alleles of chr2:55049086 (dotted line), the differential interaction map (alternate minus reference), and a CTCF ChIP‐seq track from GM12878. Color‐coded values indicate predicted chromatin interaction frequencies and differences between the reference and alternate alleles. DeepC predicts that the alternate allele leads to a loss of boundary element and affects local chromatin interactions.

A total of 365 high‐confidence and deleterious discordant variants were located within nonprotein‐coding regions. Of these, 148 variants were predicted to have likely disruptive effects on TF binding based on a RegulomeDB score of 2 or lower [38] (Figure 1). An additional three variants were predicted by the deepC model to perturb local chromatin architecture (Figure 3 and Figure S4).

Together, these 151 prioritized noncoding variants were retained for downstream analyses. Among them, 52 variants were located within putative craniofacial enhancers at one or more CSs. Using gnomAD, five variants met the prespecified global frequency criterion (MAF < 0.01) and their corresponding African/African American allele frequencies are also reported (Figure 1 and Table 2). The remaining 47 were annotated as common variants as their allele frequencies are greater than 0.01 globally (Figure 1 and Table S4).

**Table 2:** List of putative craniofacial enhancers harboring discordant deleterious rare noncoding variants that are either predicted to be functional by RegulomeDB or predicted by deepC to disrupt local chromatin architecture.

Putative craniofacial enhancer	Variants	Ref/Alt	MAF (Global)	MAF (African)	CADD score	RegulomeDB	DeepC (average score)	Nearby CF gene	ABC (NHEK)	Twin set
chr9:114286920–114289120	9:114289090	C/T	0.009866	0.174482	16.47	2b	0.000187	*COL27A1*	NIL	Twin 1
chr9:214507–215566	9:215329	G/A	0.00563	0.108276	17.77	1f	N/A	NIL	NIL	Twin 2
chr13:19861036–19865976	13:19863435	C/T	0.008581	0.030169	15.94	2b	0.001622	*MPHOSPH8*	*MPHOSPH8*	Twin 2
chr16:88381501–88384534	16:88382948	C/G	0.007368	0.025275	15.59	2a	0.003228	NIL	NIL	Twin 2
chr2:55045248–55052196	2:55049086	C/A	0.001081	0.017724	18.22	4	0.013783	*SPTBN1*	NIL	Twin 1

#### 3.3.1. Rare Discordant Variants in Noncoding Regions

The five rare variants (chr9:114289090C>T, chr9:215329G>A, chr13:19863435C>T, chr16:88382948C>G, and chr2:55049086C>A) are in the following putative enhancer regions: chr9:114286920–114289120, chr9:214507–215566, chr13:19861036–19865976, chr16:88381501–88384534, and chr2:55045248–55052196, respectively.

The chr2:55049086C>A is predicted by deepC to lead to a loss of boundary element and affects local chromatin interactions (Figure 3).

**Figure 4:**
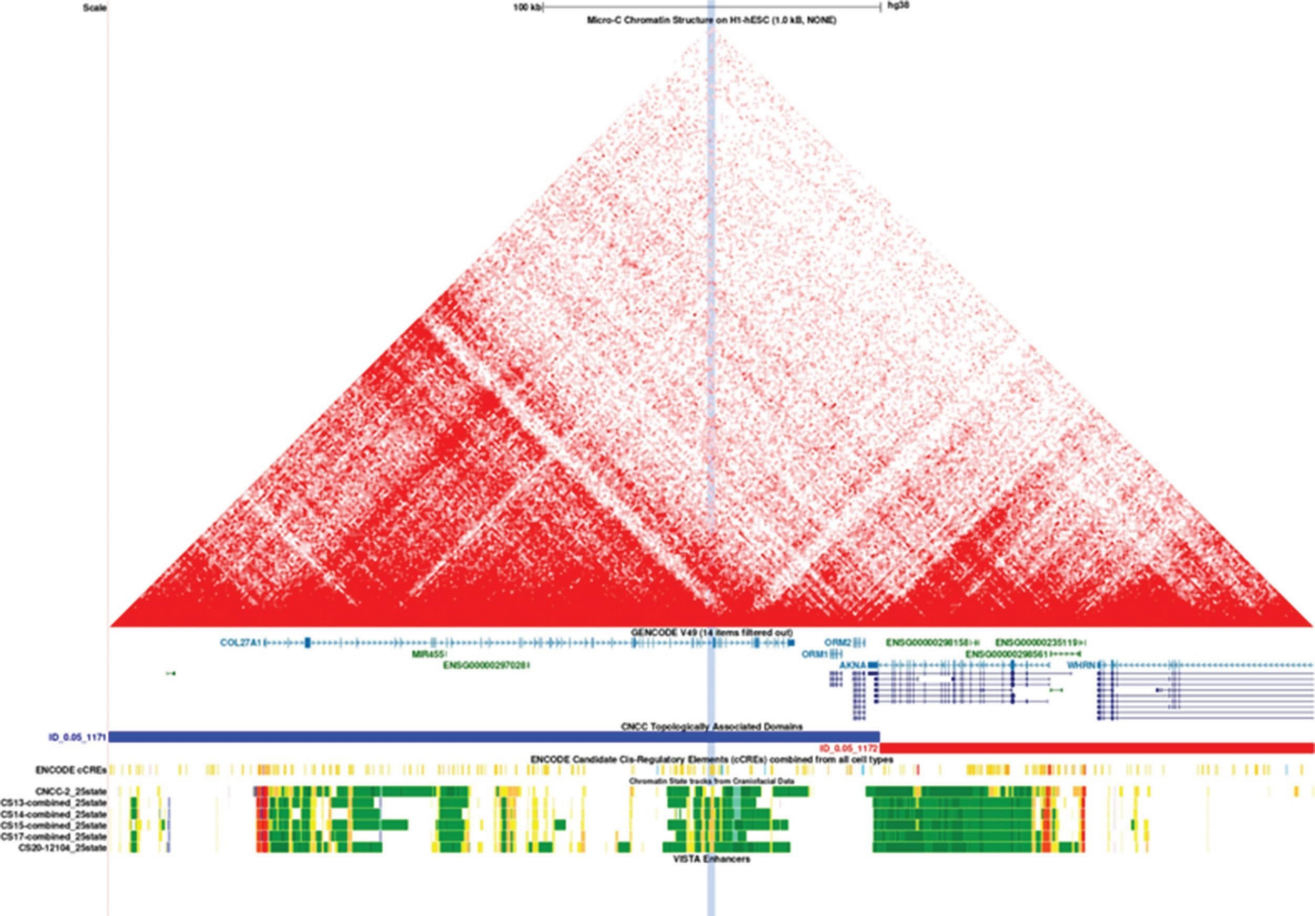
UCSC Genome Browser (GRCh38) view of the genomic region encompassing the putative enhancer at chr9:114286920–114289120. The enhancer, shown as a centrally positioned vertical blue bar, is located within the same topologically associated domain (TAD) as the *COL27A1* gene, as defined by CNCC chromatin interaction data (horizontal blue bar). Hi‐C interaction maps (red triangular matrix) indicate physical interactions between the enhancer and the *COL27A1* locus. The enhancer overlaps predicted craniofacial candidate cis‐regulatory elements (cCREs) from ENCODE and corresponds to active chromatin states (yellow) in craniofacial tissues across Carnegie stages CS13–CS20. Rao et al., Cell. (2014). Wilderman et al., Cell Rep (2018).

Three prioritized enhancers were located near genes whose perturbation has been associated with craniofacial phenotypes in mouse models. Specifically, chr9:114286920–114289120, chr13:19861036–19865976, and chr2:55045248–55052196 enhancer regions are located near *COL27A1*, *MPHOSPH8*, and *SPTBN1* genes, respectively (Figure 4, Figure S5, and Figure S6). Their positional relationships with the enhancers and their reported role in craniofacial development were used to nominate these genes as plausible candidate genes.

ABC analysis using NHEK cells, which served as a cellular proxy for embryonic oral epithelium, predicted potential enhancer‐promoter interactions involving *MPHOSPH8* and the chr13:19861036–19865976 region, which overlaps with our chr13:19863377–19863886 enhancer (ABC score = 0.021). The analysis also predicted other potential interactions with *ZMYM5* (ABC score = 1.0), *ZMYM2* (ABC score = 0.052), and *PSPC1* (ABC score = 0.042).

The chr9:114289090C>T variant located within the chr9:114286920–114289120 enhancer is predicted to disrupt binding sites for *ZNF528* and for a composite *ETV5:HOXA2* motif, while creating novel binding sites for *MYOG*, *MSC*, and *MYF5* (Table 3; Figure S7). *COL27A1*, which is near this enhancer, is predominantly expressed in ectodermal cells, with lower expression observed in mesenchymal populations (Figure S8) [40]. Interestingly, the expression pattern of *ZNF528* closely mirrors that of *COL27A1* [40]. However, *ETV5* has knockout mouse models with craniofacial phenotype [37].

**Table 3:** Transcription factors predicted by JASPAR with statistically significant gains or losses of binding in the presence of variants chr9:114289090, chr13:19863435, and chr2:55049086, located within the enhancer regions chr9:114286920–114289120, chr13:19861036–19865976, and chr2:55045248–55052196, respectively.

chr9:114289090C>T						
**TF**	**Class**	**p** **(ref)**	**p** **(Alt)**	**WT score**	**MT score**	**Joint (S) score**
*ZNF528*	C2H2 zinc finger factors	3.08E‐06	NA	0.8436	0.7813	−0.1668
*ETV5::HOXA2*	Tryptophan cluster factors; homeo domain factors	0.00082	NA	NA	NA	NA
*MYOG*	Basic helix‐loop‐helix factors (bHLH)	NA	0.00039	0.7035	0.8262	0.3010
*MSC*	Basic helix‐loop‐helix factors (bHLH)	NA	0.0011	0.7460	0.8540	0.2953
*MYF5*	Basic helix‐loop‐helix factors (bHLH)	NA	0.0013	0.6922	0.8247	0.3218
*STAT1::STAT2*	STAT domain factors	0.00047	0.00022	NA	NA	NA
*ZNF530*	C2H2 zinc finger factors	8.9E−06	0.000015	0.6778	0.6442	−0.0551
**chr13:19863435 C>T**						
**TF**	**Class**	**p** **(ref)**	**p** **(Alt)**	**WT score**	**MT Score**	**Joint (S) Score**
*KLF5*	C2H2 zinc finger factors	0.00041	NA	0.8578	0.8554	−0.0069
*KLF16*	C2H2 zinc finger factors	0.0011	NA	0.7900	0.7596	−0.0679
*SP3*	C2H2 zinc finger factors	0.00045	NA	0.7924	0.7725	−0.0448
*KLF11*	C2H2 zinc finger factors	0.00065	NA	0.8041	0.7762	−0.0653
*SP9*	C2H2 zinc finger factors	0.00029	NA	0.8324	0.7644	−0.1743
*ZNF148*	C2H2 zinc finger factors	6.1E−05	NA	0.8664	0.7575	−0.3123
*GFI1*	C2H2 zinc finger factors	NA	0.00087	0.7241	0.8073	0.1945
*ZNF708*	C2H2 zinc finger factors	NA	0.00017	0.7516	0.8133	0.1481
*ZNF740*	C2H2 zinc finger factors	0.00026	0.00028	0.8863	0.8863	0
*MZF1*	C2H2 zinc finger factors	0.00027	0.00030	0.8384	0.8343	−0.0109
*HINFP*	C2H2 zinc finger factors	0.0008	0.0013	0.7221	0.6373	−0.1543
*EGR3*	C2H2 zinc finger factors	0.00003	0.0003	0.8411	0.7664	−0.1974
*EGR4*	C2H2 zinc finger factors	6.75E−05	0.00047	0.8173	0.7667	−0.1234
*MAZ*	C2H2 zinc finger factors	6E−05	0.0012	0.9043	0.7827	−0.4059
*ZNF135*	C2H2 zinc finger factors	5.4E−05	0.0003	0.8287	0.7977	−0.0790
*ZNF460*	C2H2 zinc finger factors	1.44E−06	5.14E−06	0.8611	0.8363	−0.0718
*SP5*	C2H2 zinc finger factors	5.1E−05	0.00015	0.9680	0.9418	−0.1367
**chr2:55049086C>A**						
**TF**	**Class**	**p** **(ref)**	**p** **(Alt)**	**WT score**	**MT Score**	**Joint (S) score**
*HSF1*	Heat shock factors	0.00022	NA	0.8384	0.8373	−0.0029
*ZBTB12*	C2H2 zinc finger factors	0.0012	NA	0.8140	0.8028	−0.0271
*PRDM1*	C2H2 zinc finger factors	0.00034	NA	0.7949	0.8099	0.0358
*KLF5*	C2H2 zinc finger factors	0.00077	NA	0.7962	0.7962	0
*FOXJ2::ELF1*	Fork head/winged helix factors; Tryptophan cluster factors	0.00124	NA	NA	NA	NA
*HSF2*	Heat shock factors	0.0002	NA	0.8057	0.8192	0.0333
*HSF4*	Heat shock factors	0.00019	NA	0.8398	0.8732	0.1017
*ZBTB26*	C2H2 zinc finger factors	0.0014	NA	0.7944	0.7981	0.0085
*NR1I3*	Nuclear receptors with C4 zinc fingers	NA	0.003	0.7955	0.7981	0.0060
*E2F1*	Fork head/winged helix factors	NA	0.0026	0.6499	0.6499	0
*SMAD3*	SMAD/NF‐1 DNA‐binding domain factors	NA	0.0012	0.5775	0.5775	0
*NFIX*	SMAD/NF‐1 DNA‐binding domain factors	NA	0.0009	0.7617	0.7617	0
*E2F2*	Fork head/winged helix factors	NA	0.0015	0.6019	0.5723	−0.0412
*SP2*	C2H2 zinc finger factors	NA	0.0017	0.7444	0.7234	−0.0409
*KLF14*	C2H2 zinc finger factors	NA	0.0029	0.6817	0.6788	−0.0048
*SMAD5*	SMAD/NF‐1 DNA‐binding domain factors	NA	0.0029	0.6208	0.5890	−0.0460
*TBX3*	T‐box factors	NA	0.0021	0.7098	0.6779	−0.0566
*ZBTB14*	C2H2 zinc finger factors	NA	0.00048	0.5500	0.5456	−0.0055

Similarly, the chr13:19863435C>T variant within the chr13:19861036–19865976 enhancer is predicted to disrupt binding sites for *KLF5*, *KLF16*, *SP3*, *KLF11*, *SP9*, and *ZNF148*, while creating novel binding sites for *GFI1* and *ZNF708* (Table 3; Figure S7). *MPHOSPH8*, located near this enhancer, is primarily expressed in blood cell populations (Figure S9) [40]. Among the affected TFs, *SP3*, which has mouse models with craniofacial phenotype shows an expression pattern that somewhat mirrors the expression pattern of *MPHOSPH8* [40]. Interestingly, both *SP3* and *MPHOSPH8* exhibit high expression at CS16, with *MPHOSPH8* also expressed at CS20. Consistent with this temporal pattern, the chr13:19861036–19865976 enhancer is annotated as accessible at CS18 and 19. In addition, *KLF5*, which has been implicated in craniofacial development in mice, is expressed at CS20, corresponding to the later stage of *MPHOSPH8* expression [37] (Figure S10).

Finally, the chr2:55049086C>A variant within the chr2:55045248–55052196 enhancer is predicted to both disrupt existing TFBS and create novel binding sites for TFs with implication in craniofacial development (Table 3; Figures S7 and S11).

#### 3.3.2. Common‐Frequency Discordant Variants in Noncoding Regions

Among the 47 common variants, 11 variants had been previously associated with noncraniofacial traits in genome‐wide association studies (GWAS). These traits included abnormalities of the skeletal system (rs578347), kidney volume (rs271762), intraocular pressure (rs76070960), dementia (rs1460072), polyunsaturated fatty acid levels (rs6937088), platelet‐related traits (rs4145343, rs13287061, and rs1005165), atrial fibrillation (rs7819550), bipolar disorder or major depressive disorder (rs117763335), and C‐reactive protein levels (rs6519133). Eight of these GWAS‐linked variants were located within enhancers proximal to genes with craniofacial phenotypes including *KIF1B*, *SPTBN1*, *TLL1*, *SCUBE3*, *SOSTDC1*, *RDH10*, *ADAMTSL2*, and *OPA3* (Table S5).

It is interesting to note that the putative enhancer (chr1:10662943–10683543) near *KIF1B* overlaps with a functionally tested enhancer (VISTA ID: hs289.0) which is active the neural tube of mice at E11.5 (Figure S12) [47].

JASPAR analysis of the eight GWAS‐linked variants predicts disruption of existing TFBS as well as the creation of novel binding sites for TFs previously implicated in craniofacial development in mouse models (Table S6).

### 3.4. Strongest Candidate Loci With Convergent Evidence

The 37,271 discordant variants identified between the affected and unaffected MZ twins were distributed across both protein‐coding and noncoding regions, with subsequent prioritization identifying candidates with diverse potential functional consequences. Overall, the findings did not point to a single genetic locus or mechanism underlying phenotypic discordance. Instead, the prioritized variants encompassed both predicted protein‐altering variants and regulatory variants predicted to affect putative craniofacial enhancers, suggesting heterogeneous genetic mechanisms. Among the coding variants, the *PYGB* and *PCDHA8* variants represented the strongest candidate based on their predicted effect on protein structure and *PYGB* expression in embryonic craniofacial tissues. Among the noncoding variants, the three rare variants in enhancers near *SPTBN1*, *COL27A1*, and *MPHOSPH8* represented the strongest candidates based on multiple converging features that include predicted disruption of TFBS and proximity to genes with reported craniofacial phenotypes in mouse models.

## 4. Discussion

Phenotypic discordance between MZ twins may arise through sequence variation and other mechanisms such as tissue‐specific mosaicism, epigenetic differences, and environmental or developmental factors [48]. In this study, we used whole‐genome sequencing of two African‐ancestry MZ twin pairs discordant for nsOFC to identify and prioritize sequence variants that may contribute to phenotypic discordance. Our analysis identified candidate discordant variants across both protein‐coding and noncoding regulatory regions. These findings support a model in which discordant sequence variation represents one possible contributor to phenotypic discordance, potentially through effects on protein function or developmental gene regulation. To our knowledge, this is the first study to identify discordant variants among MZ twin pairs of African ancestry with nsOFC.

### 4.1. Contribution of Discordant Variants in the Protein‐Coding Region

Our analysis of discordant coding variants identified coding variants predicted to have potentially damaging effects on *PYGB* and *PCDHA8* genes. *PYGB* plays a central role in cellular energy metabolism by catalyzing the degradation of glycogen to glucose‐1‐phosphate, thereby supporting adenosine triphosphate production required for energy‐dependent cellular processes such as muscle contraction [49]. Previous studies have demonstrated that *PYGB* is involved in multiple physiological and pathological processes, particularly in tissues that require rapid energy availability, and it is broadly expressed across many tissue types [49]. SysFACE‐based expression analysis shows that it is expressed in the mouse maxilla and frontonasal regions.

The *PCDHA8* gene, which is a member of the protocadherin family, plays key roles in cell adhesion, neurite initiation and outgrowth, axon guidance and fasciculation, as well as synapse formation and stabilization [50]. Cell adhesion plays an essential role in cranial and facial development, and mutations in cell‐to‐cell adhesion genes have been shown to disrupt normal craniofacial morphogenesis, particularly contributing to cleft lip with or without cleft palate [51]. However, to date, there is no evidence of *PCDHA8* expression in craniofacial tissues, nor have mouse models with alterations in this gene been associated with craniofacial defects.

Similarly, the prioritized splice‐site variants were in genes without established roles in craniofacial development.

Taken together, these findings are consistent with previous reports suggesting that highly penetrant coding variants are relatively uncommon contributors to clefting [5, 18, 52] and are unlikely to fully account for phenotypic discordance in most MZ twin pairs. Nonetheless, the potential involvement of these variants cannot be totally excluded.

### 4.2. Contribution of Rare Discordant Variants in the Noncoding Region

A substantial proportion of the prioritized discordant variants identified in this study were in noncoding regions, including putative craniofacial enhancers. However, this high proportion of prioritized noncoding variants should not, by itself, be interpreted as evidence that noncoding variation contributes more strongly than coding variation to phenotypic discordance, given that noncoding regions comprise most of the human genome.

Among the five rare variants located within putative craniofacial enhancers, three are near genes whose perturbation have been associated with craniofacial phenotypes in mouse models: *COL27A1*, *MPHOSPH8*, and *SPTBN1*.

*COL27A1* encodes the pro‐alpha chain of fibrillar collagen type XXVII, which is primarily expressed in cartilage and is thought to play a role in transition of cartilage to bone [53]. Mice carrying homozygous deletion of 87 amino acids in *Col27a1* exhibit severe chondrodysplasia with midline defects and die shortly after birth because of respiratory failure [54]. When this deletion was restricted to chondrocytes, homozygous mice survived postnatally but were significantly smaller and displayed craniofacial abnormalities including a shortened snout and domed skull among other phenotypes [55]. In chondrocyte studies, expression of human *COL27A1* is regulated by the TF *SOX9*, which is a key regulator of vertebrate chondrogenesis [56]. In contrast, our analysis of putative craniofacial enhancers implicated a nearby enhancer in which its harbored variant is predicted to lead to loss of binding sites for the TFs *ZNF528* and for a composite *ETV5:HOXA2* motif and the creation of novel binding sites for *MYOG*, *MSC*, and *MYF5*. The expression pattern of *ZNF528* closely mirrors that of *COL27A1* [40]. Although *ETV5* has not been directly linked to clefting, it has been functionally implicated in abnormal vomeronasal organ morphology in mouse models, providing evidence for potential regulatory involvement at this locus that requires experimental validation [37].

*MPHOSPH8* encodes M‐phase phosphoprotein 8 (MPP8) [57]. Full‐body knockout of *Mphosph8* results in embryonic lethality, with complete loss leading to 100% lethality before embryonic day 11.5 [57]. Nervous system–specific deletions of *Mphosph8* resulted in markedly increased brain‐to‐body ratios [57]. Our JASPAR analysis of variants within a putative craniofacial enhancer near *MPHOSPH8* predicted loss of binding sites for *KLF5*, *KLF16*, *SP3*, *KLF11*, *SP9,* and *ZNF148*, along with the creation of novel binding sites for *GFI1* and *ZNF708*. Among the affected TFs, *SP3* showed an expression pattern that partially mirrored that of *MPHOSPH8* [40]. Notably, *SP3* has been associated with abnormal cranial and nasal bone morphology in mouse models, further supporting its potential relevance in craniofacial development [37].

*SPTBN1* encodes beta II spectrin, a ubiquitously expressed cytoskeletal protein that forms membrane‐associated networks. In mice, *Sptbn1* variants are associated with a broad spectrum of craniofacial abnormalities including facial dysmorphism, microcephaly, and macrocephaly [58]. JASPAR analysis of the variant within the putative craniofacial enhancer near *SPTBN1* predicted both loss and gain of TFBS. Several of these TFs, including *PRDM1*, *KLF5*, *NFIX*, *SMAD5*, and *TBX3*, have been linked to craniofacial defects in mouse models. In addition, the variant in the enhancer was predicted by deepC to lead to a loss of boundary element. These predictions raise the possibility that the variant could affect gene regulation through altered TF binding and/or chromatin organization.

### 4.3. Contribution of Common Discordant Variants in the Noncoding Region

In addition to the rare variants, we identified common discordant variants within putative craniofacial enhancers that have previously been associated with noncraniofacial traits in GWAS. Although several of these variants are in or near regulatory regions with potential relevance to craniofacial genes, their prior GWAS associations do not provide evidence that they contribute to OFC or craniofacial development. These observations are hypothesis‐generating and suggest potential pleiotropic regulatory effects that require independent functional validation

One particular example is the enhancer chr1:10662943–10683543, which overlaps a functionally validated VISTA enhancer, hs289.0. This enhancer drives LacZ reporter expression in the neural tube of mice at embryonic day 11.5 [47]. The enhancer lies in proximity to *KIF1B*, a gene for which mouse models display abnormal facial morphology [37]. However, this proximity does not establish *KIF1B* as the target gene of the enhancer. *KIF1B* encodes a kinesin‐like motor protein that is predominantly expressed in neurons and glial cells and plays a well‐established role in synaptic vesicle transport and axonal outgrowth [59]. The common variant within this enhancer is predicted to disrupt binding sites for *SP3* and *SP8*. *SP8* has been implicated in mouse models of cleft palate and cleft lip, whereas *SP3* has been associated with abnormal cranial and nasal bone morphology [37]. Disruption of binding for these TFs provides a computationally predicted regulatory mechanism that could be investigated experimentally in the context of craniofacial development.

### 4.4. A Proposed Overarching Model of Candidate Genetic Mechanisms Underlying Phenotypic Discordance

Our findings support a model in which phenotypic discordance between MZ twins may arise from multiple classes of discordant sequence variants acting through distinct but potentially convergent mechanisms (Figure S13). Both twins are expected to share the majority of their inherited genetic background, which may establish a baseline susceptibility to nsOFC. Against this shared background, sequence variants that differ between the affected and unaffected co‐twins could contribute additional genetic liability. In our study, these candidate variants included both protein‐coding and noncoding regulatory variants. However, sequence variation represents only one potential source of discordance; epigenetic differences, tissue‐specific mosaicism, and environmental or developmental factors may also contribute. Importantly, this model represents a conceptual framework rather than evidence of a causal pathway. Functional validation and analysis of additional discordant twin pairs will be required to determine whether the candidate variants identified here contribute directly to phenotypic discordance.

## 5. Limitations

This study has several important limitations. First, only two MZ twin pairs were available, substantially limiting statistical power and preventing formal enrichment analyses or assessment of recurrence of candidate variants across multiple families. Consequently, the findings should be considered exploratory and hypothesis‐generating. Second, the prioritized variants were supported by sequence‐level and computational evidence, but independent experimental validation was not performed. Third, the regulatory annotations relied on publicly available datasets and computational models generated from tissues or cell types that may not fully recapitulate the developmental context of human craniofacial morphogenesis.

## 6. Conclusions

Whole genome sequencing of two MZ twin pairs of African ancestry discordant for nsOFC identified candidate discordant variants in both protein‐coding and noncoding regions. Several variants were predicted to affect protein function, TF binding, or chromatin interactions, including variants within putative craniofacial enhancers near *COL27A1*, *MPHOSPH8*, and *SPTBN1*. These findings support the possibility that discordant sequence variation may contribute to phenotypic discordance in MZ twins with nsOFC, while recognizing that epigenetic differences and environmental or developmental factors may also contribute. The prioritized variants provide candidate loci for future functional validation and investigation in additional discordant twin pairs.

## Author Contributions

Aladenika E. and Butali A. contributed to the conception and design, data acquisition and curation, analysis, interpretation of results, draft writing, and critically revised the manuscript. Olujitan M., Busch T., Gowans L., Adeyemo W., Adekunle A., Mekonen E., Ajala O., Gadde R., Mba N., and Azeez A. contributed to data acquisition, analysis and interpretation of results, and critically revised the manuscript.

## Funding

This study was supported by the National Institute of Dental and Craniofacial Research (NIDCR) (10.13039/100000002; R01‐DE028300; 03‐DE035068).

## Disclosure

All authors gave their final approval and agreed to be accountable for all aspects of the work.

## Ethics Statement

Ethical approval was obtained from review boards at the Lagos University Teaching Hospital (ADM/DCST/HREC/VOL.XV/321), Obafemi Awolowo University Teaching Hospital (ERC/2011/12/01), Kwame Nkrumah University of Science and Technology (CHRPE/RC/018/130), University of Iowa (IRB ID #: 201101720).

## Conflicts of Interest

The authors declare no conflicts of interest.

## Supporting information


**Supporting Information** Additional supporting information can be found online in the Supporting Information section. Figure S1: Pedigrees of the two monozygotic twin pairs in our study. Twin pair 1 is from Ghana and twin pair 2 is from Nigeria. Twin 1A has unilateral cleft lip on the left side. Twin 2A has unilateral cleft lip and palate on the left side. Table S1: Pairwise identity‐by‐descent (IBD) probabilities between the 2 sets of monozygotic twins. Table S2: Read depth, reference and alternate read counts, genotype quality, and variant allele fraction for prioritized discordant variants. Table S3: List of discordant deleterious variants in coding regions. Figure S2: SysFACE‐based expression analysis of *PYGB* gene during mouse facial development. The intensity of the red color in the heatmap represents the level of candidate gene expression. Figure S3: Structural impact of the discordant variant in the *PCDHA1* gene as predicted by HOPE. The variant residue is smaller than and less hydrophobic than the corresponding WT residue, which may affect local protein stability or interactions. Figure S4: Predicted impact of the variants chr16:29082111 (panel A) and chr6:36660895 (panel B) using the deepC model trained in GM12878 cells. Shown are deepC predictions for the reference and alternate alleles (dotted line), the differential interaction map (alternate minus reference), and a CTCF ChIP–seq track from GM12878. Color‐coded values indicate predicted chromatin interaction frequencies and differences between the reference and alternate alleles. DeepC predicts that the alternate alleles disrupt a boundary‐forming effect. Table S4: List of discordant deleterious common noncoding variants predicted to be functional by RegulomeDB or predicted by deepC to disrupt local chromatin architecture. Figure S5: UCSC Genome Browser (GRCh38) view of the genomic region containing a putative enhancer at chr13:19861036–19865976. The enhancer (vertical blue bar) resides within the same topologically associated domain (TAD) as the *MPHOSPH8* gene. Hi‐C contact maps (red triangular matrix) reveal chromatin interactions between the enhancer and the *MPHOSPH8* gene. Figure S6: UCSC Genome Browser (GRCh38) view of the genomic region harboring a putative enhancer at chr2:55045248–55052196. The enhancer (vertical blue bar) is located within the same topologically associated domain (TAD) as the *SPTBN1* gene, as supported by CNCC chromatin interaction data (horizontal blue bar). Hi‐C interaction maps (red triangular matrix) demonstrate physical contacts between the enhancer and the *SPTBN1* gene. Figure S7: Sequence logos representing binding sites for transcription factors that are predicted to be altered (loss and gain) by the variants in the prioritized putative craniofacial enhancers: (A) ZNF528, (B) KLF16, (C) KLF5, (D) SMAD5, and (E) SBTB14. Figure S8: Single‐nucleus RNA‐seq co‐projection of human craniofacial tissues at CS17 showing the expression of *COL27A1* and transcription factors affected by variants within the enhancer at chr9:114286920–114289120. (A) UMAP visualization depicting *COL27A1* expression across distinct craniofacial cell populations. (B) Heatmap illustrating scaled gene expression by cell type for *COL27A1* and transcription factors predicted to gain or lose binding sites due to enhancer‐associated variants. Figure S9: Single‐nucleus RNA‐seq projection of human craniofacial tissue illustrating the expression of *MPHOSPH8* and transcription factors impacted by chr13:19861036–19865976 enhancer‐associated variant. (A) UMAP representation showing *MPHOSPH8* expression across distinct cell populations. (B) Heatmap displaying scaled gene expression by cell type for *MPHOSPH8* and transcription factors predicted to gain or lose binding sites because of variants within the enhancer region chr13:19861036–19865976. Figure S10: Heatmap displaying scaled gene expression by Carnegie stage for *MPHOSPH8* and transcription factors predicted to gain or lose binding sites because of variants within the enhancer region chr13:19861036–19865976. Figure S11: Single‐nucleus RNA‐seq projection of human craniofacial tissue highlighting the expression of *SPTBN1* and transcription factors influenced by chr2:55045248–55052196 enhancer variants. (A) UMAP visualization of *SPTBN1* expression across craniofacial cell populations. (B) Heatmap showing scaled gene expression by cell type for *SPTBN1* and transcription factors predicted to gain or lose binding sites due to variants within the enhancer region chr2:55045248–55052196. Table S5: Discordant deleterious common noncoding variants predicted to be functional or to alter local chromatin architecture and previously reported as genome‐wide significant with any trait in the GWAS Catalog Figure S12: LacZ reporter activity observed in the neural tube of mouse embryos following random transgenesis driven by the VISTA enhancer element hs289.0 (chr1:10672013–10673061). This enhancer overlaps with our putative craniofacial enhancer region chr1:10662943–10683543. Table S6: Transcription factors predicted by JASPAR to show statistically significant gains or losses of binding in the presence of deleterious common noncoding variants that are predicted to be functional or to alter local chromatin architecture and that have been previously reported as genome‐wide significant for at least one trait in the GWAS Catalog. Figure S13: A proposed overarching genetic mechanism underlying phenotypic discordance in monozygotic twins.

## Data Availability

The data that support the findings of this study are available on request from the corresponding author. The data are not publicly available due to privacy or ethical restrictions.
