# Supplementary material for: Whole Genome Sequencing of Discordant Monozygotic Twins Reveals Regulatory Variants Associated With Nonsyndromic Orofacial Clefts
Source: Hum Mutat. 2026 Sep 23;2026:2471705. doi: 10.1155/humu/2471705 (PMC13601850; doi:10.1155/humu/2471705)
Supplement: Supplementary file 1 [file HUMU-2026-2471705-s001.docx]

**Figure S1:** Pedigrees of the two monozygotic twin pairs in our study. Twin pair 1 is from Ghana and Twin pair two is from Nigeria. Twin 1A has unilateral cleft lip on the left side. Twin 2A has unilateral cleft lip and palate on the left side.

**Table S1:** Pairwise identity-by-descent (IBD) probabilities between the 2 sets of monozygotic twins

| **Individual ID** | **Individual ID** | **Z0** | **Z1** | **Z2** |
| --- | --- | --- | --- | --- |
| Twin1A | Twin1B | 0 | 0.0001 | 0.9999 |
| Twin2A | Twin2B | 0 | 0.0001 | 0.9999 |

Z0, Z1, and Z2 represent the probabilities that a pair of individuals share zero, one, or two alleles identical by descent, respectively.

**Table S2**: Read depth, reference and alternate read counts, genotype quality, and variant allele fraction for prioritized discordant variants

| Genomic region | Variant | Ref/Alt | Genotype quality | Read Depths | Ref reads | Alt reads | Variant Allele Frequency (Fraction) | Twin pair |
| --- | --- | --- | --- | --- | --- | --- | --- | --- |
| Coding region | 5:140842949 | C/A | 41 | 32 | 17 | 15 | 0.46875 | Twin 1 |
|  | 20:25278370 | G/T | 43 | 35 | 16 | 19 | 0.542857 | Twin 2 |
|  | 7:121267212 | C/A | 45 | 26 | 11 | 15 | 0.576923 | Twin 1 |
|  | 7:67278874 | G/A | 20 | 17 | 7 | 10 | 0.588235 | Twin 2 |
|  | 8:33513081 | C/T | 48 | 43 | 21 | 22 | 0.511628 | Twin 1 |
|  | 17:20501924 | C/G | 24 | 33 | 19 | 14 | 0.424242 | Twin 2 |
|  | 19:28727576 | T/C | 45 | 28 | 12 | 16 | 0.571429 | Twin 1 |
| Non-coding region | 9:114289090 | C/T | 48 | 25 | 0 | 25 | 1 | Twin 1 |
|  | 9:215329 | G/A | 43 | 21 | 7 | 14 | 0.666667 | Twin 2 |
|  | 13:19863435 | C/T | 40 | 14 | 8 | 6 | 0.428571 | Twin 2 |
|  | 16:88382948 | C/G | 31 | 11 | 5 | 6 | 0.545455 | Twin 2 |
|  | 2:55049086 | C/A | 44 | 21 | 11 | 10 | 0.47619 | Twin 1 |

**Table S3:** List of discordant deleterious variants in coding regions

| Variant | Ref/Alt | Gene | Effect | MAF (Global) | MAF (Africans) | CADD score | SIFT | PolyPhen | SpliceAI | Twin pair |
| --- | --- | --- | --- | --- | --- | --- | --- | --- | --- | --- |
| 2:217848559 | G/A | TNS1 | Missense | 0.32912 | 0.56298 | 22.4 | 0.03 | 0.452 | - | Twin 1 |
| 3:8635815 | G/A | SSUH2 | Missense | 0.004032 | 0.07028 | 24 | 0.03 | 0.998 | - | Twin 1 |
| 3:150410161 | G/A | TSC22D2 | Missense | 0.005549 | 0.103879 | 17.15 | 1 | 0.631 | - | Twin 1 |
| 3:155763651 | T/G | C3orf33 | Missense | 0.003537 | 0.064316 | 16.99 | 0.03 | 0.006 | - | Twin 1 |
| 3:194341097 | C/T | CPN2 | Missense | 0.284494 | 0.176218 | 19.23 | 0.01 | 0.993 | - | Twin 1 |
| 3:158732380 | C/G | RARRES1 | Missense | 0.181549 | 0.283844 | 19.43 | 0.02 | 0.522 | - | Twin 2 |
| 4:122192273 | A/G | BLTP1 | Missense | 0.036679 | 0.050124 | 18.58 | 0 | 0.031 | - | Twin 2 |
| 4:153592475 | A/G | TMEM131L | Missense | 0.308102 | 0.174875 | 15.18 | 1 | 0 | - | Twin 2 |
| 5:108380859 | G/A | FBXL17 | Missense | 0.001877 | 0.02868 | 17.21 | 0.06 | 0.092 | - | Twins 1 |
| 5:140842949 | C/A | PCDHA8 | Missense | 0.003256 | 0.060805 | 20.6 | 0 | - | - | Twins 1 |
| 5:176532045 | C/T | RNF44 | Missense | 0.00052 | 0.009421 | 22.3 | 0.07 | 0.494 | - | Twins 1 |
| 6:33069222 | T/C | HLA-DPA1 | Missense | 0.2112 | 0.499598 | 19.07 | 0.2 | 0.106 | - | Twin 1 |
| 7:121267212 | C/A | CPED1 | Splice site (acceptor) | 0.001662 | 0.027117 | 19.18 | - | - | 0.31 | Twin 1 |
| 7:149455769 | G/A | ZNF777 | Missense | 0.003171 | 0.059779 | 16.84 | 0.01 | 0.015 | - | Twin 1 |
| 7:7381549 | G/T | COL28A1 | Missense | 0.002951 | 0.055879 | 17.84 | 0.12 | 0.129 | - | Twin 2 |
| 7:67278874 | G/A | PMS2P4 | Splice site (donor) | 0.092093 | 0.059184 | 17.61 | - | - | 0.96 | Twin 2 |
| 7:130733862 | G/A | KLF14 | Missense | 0.797516 | 0.625288 | 20.2 | 0.14 | 0.81 | - | Twin 2 |
| 8:8376703 | G/A | PRAG1 | Missense | 0.33103 | 0.355551 | 22.4 | 0.05 | 0.559 | - | Twin 1 |
| 8:33513081 | C/T | TTI2 | Splice site (donor) | 0.014697 | 0.053067 | 21.9 | - | - | 0.94 | Twin 1 |
| 8:120502582 | C/G | MTBP | Missense | 0.002719 | 0.053592 | 17.82 | 0.02 | 0.007 | - | Twin 1 |
| 9:136722290 | A/G | DIPK1B | Missense | 0.93871 | 0.609771 | 15.47 | 0.18 | 0 | - | Twin 1 |
| 9:137614850 | G/T | ARRDC1 | Missense | 0.096886 | 0.066485 | 18.2 | 0.03 | 0.543 | - | Twin 1 |
| 9:1051758 | G/C | DMRT2 | Missense | 0.000397 | 0.008021 | 24.9 | 0.01 | 0 | - | Twin 2 |
| 9:35829393 | C/T | TMEM8B | Splice site | 0.601539 | 0.548499 | 20.4 | - | - | - | Twin 2 |
| 10:50125432 | G/T | WASHC2A | Missense | 0.001915 | 0.038077 | 23.8 | 0.02 | 0.957 | - | Twin 1 |
| 10:86962641 | A/T | SNCG | Stop lost | 0.236347 | 0.186844 | 17.1 | 0.04 | - | - | Twin 2 |
| 11:199492 | G/A | ODF3 | Missense | 0.070106 | 0.047913 | 24.3 | 0.16 | 0.773 | - | Twin 1 |
| 11:17517424 | C/T | USH1C | Missense | 0.003066 | 0.05143 | 21.4 | 0.36 | - | - | Twin 1 |
| 11:67282821 | G/A | GRK2 | Splice site (donor) | 0.880149 | 0.296097 | 16.09 | - | - | 0 | Twin 1 |
| 11:134282081 | G/C | GLB1L3 | Missense | 0.97 | 0.974601 | 18.45 | 1 | 0 | - | Twin 1 |
| 11:65641466 | C/T | SIPA1 | Missense | 0.143944 | 0.267063 | 20.4 | 0.01 | 0.198 | - | Twin 2 |
| 12:6019732 | A/C | VWF | Missense | 0.002237 | 0.016044 | 16.85 | 1 | 0 | - | Twin 1 |
| 12:68932767 | C/T | CPM | Missense | 0.042479 | 0.511869 | 19.6 | 0.88 | 0 | - | Twin 1 |
| 12:107319919 | G/A | ABTB3 | Missense | 0.495947 | 0.246728 | 16.34 | 0.07 | 0 | - | Twin 1 |
| 12:52792304 | G/C | KRT3 | Missense | 0.614865 | 0.413075 | 18.55 | 0 | 0.904 | - | Twin 2 |
| 13:42048793 | A/C | DGKH | Missense | 0.00534 | 0.090194 | 23.5 | 0.05 | 0 | - | Twin 2 |
| 14:39431808 | G/A | FBXO33 | Missense | 0.003075 | 0.056691 | 17.38 | 0.73 | 0 | - | Twin 1 |
| 15:83067087 | G/A | BTBD1 | Missense | 0.003191 | 0.042432 | 18.58 | 0.05 | 0 | - | Twin 1 |
| 15:88858758 | C/T | ACAN | Splice site | 0.0053 | 0.099611 | 18.71 | 0 | 0.024 | - | Twin 1 |
| 15:55498216 | G/T | DNAAF4 | Missense | 0.01014 | 0.198406 | 18.88 | 0.05 | 0.127 | - | Twin 2 |
| 16:57701988 | G/C | DRC7 | Missense | 0.267025 | 0.262634 | 23.3 | 0.05 | 0.124 | - | Twin 1 |
| 16:81198985 | T/C | PKD1L2 | Missense | 0.00569 | 0.051964 | 19.06 | 0.02 | 0.999 | - | Twin 2 |
| 16:88712724 | C/T | CTU2 | Missense | 0.113675 | 0.221954 | 21.6 | 0.09 | 0.177 | - | Twin 2 |
| 17:7388788 | G/A | TNK1 | Missense | 0.906033 | 0.518639 | 21.6 | 0.04 | 0.996 | - | Twin 1 |
| 17:18672398 | G/A | FOXO3B | Missense | 0.002929 | 0.056944 | 25 | - | - | - | Twin 1 |
| 17:18672848 | G/C | FOXO3B | Missense | 0.004828 | 0.0926 | 24.6 | - | - | - | Twin 1 |
| 17:41117835 | G/C | KRTAP4-11 | Missense | 0.034592 | 0.111691 | 17.26 | 0.07 | 0 | - | Twin 1 |
| 17:43953160 | A/G | PYY | Missense | 0.00572 | 0.105686 | 17.33 | 0 | 0.472 | - | Twin 1 |
| 17:20501924 | C/G | KRT16P3 | Splice site (donor) | 0.005914 | 0.059718 | 15.62 | - | - | 0.78 | Twin 2 |
| 17:41160589 | A/T | KRTAP4-4 | Missense | 0.018103 | 0.338647 | 19.31 | 0.01 | 0 | - | Twin 2 |
| 17:41734561 | C/T | HAP1 | Missense | 0.002356 | 0.037885 | 16.52 | 0.04 | 0.598 | - | Twin 2 |
| 17:80065575 | G/A | CCDC40 | Missense | 0.001182 | 0.022659 | 24.7 | 0 | 0.999 | - | Twin 2 |
| 18:9221885 | C/G | ANKRD12 | Missense | 0.077069 | 0.0476 | 19.54 | 1 | 0.029 | - | Twin 1 |
| 18:11609995 | C/T | SLC35G4 | Missense | 0.190413 | 0.353867 | 18.84 | 0.08 | 0.723 | - | Twin 1 |
| 18:11610002 | A/G | SLC35G4 | Missense | 0.188579 | 0.353233 | 16.06 | 0 | 0 | - | Twin 1 |
| 18:11610163 | G/A | SLC35G4 | Missense | 0.183399 | 0.415774 | 15.79 | 0.03 | 0 | - | Twin 2 |
| 18:11610232 | C/G | SLC35G4 | Missense | 0.004082 | 0.065363 | 18.86 | 0.01 | 0.995 | - | Twin 2 |
| 19:22664610 | G/A | ZNF492 | Missense | 0.015427 | 0.27559 | 20.6 | 0.01 | 0.929 | - | Twin 1 |
| 19:28727576 | T/C | LOC100420587 | Splice site (donor) | 0.173455 | 0.08506 | 15.29 | - | - | 0.32 | Twin 1 |
| 19:49035719 | C/A | CGB1 | Missense | 0.003485 | 0.06532 | 15.65 | 0.51 | 0.492 | - | Twin 1 |
| 19:47256616 | T/G | CCDC9 | Splice site (donor) | 0.438559 | 0.336407 | 15.37 | - | - | 0.01 | Twin 2 |
| 20:3694851 | T/G | SIGLEC1 | Missense | 0.640027 | 0.778445 | 16.58 | 1 | 0 | - | Twin 1 |
| 20:25278370 | G/T | PYGB | Missense | 0.1746 | 0.076581 | 32 | 0 | 0.815 | - | Twin 2 |
| 21:34370503 | C/G | KCNE2 | Missense | 0.000851 | 0.016247 | 17.16 | 0.3 | 0.007 | - | Twin 1 |
| 21:46124924 | G/A | COL6A2 | Splice site (donor) | 0.052066 | 0.085476 | 18.11 | - | . | 0 | Twin 2 |
| 22:20859240 | T/C | SNAP29 | Missense | 0.001549 | 0.026213 | 19.66 | 0.11 | 0.375 | - | Twin 1 |

**Figure S2:** SysFACE-based expression analysis of *PYGB* gene during mouse facial development. The intensity of the red color in the heatmap represents the level of candidate gene expression.

**Figure S3:** Structural impact of the discordant variant in the *PCDHA1* gene as predicted by HOPE. The variant residue is smaller than and less hydrophobic than the corresponding wild-type residue, which may affect local protein stability or interactions.

**Figure S4:** Predicted impact of the variants chr16:29082111 (panel A) and chr6:36660895 (panel B) using the DeepC model trained in GM12878 cells. Shown are DeepC predictions for the reference and alternate alleles (dotted line), the differential interaction map (alternate minus reference), and a CTCF ChIP–seq track from GM12878. Color-coded values indicate predicted chromatin interaction frequencies and differences between the reference and alternate alleles. DeepC predicts that the alternate alleles disrupt a boundary-forming effect.

**Table S4:** List of Discordant deleterious common noncoding variants predicted to be functional by RegulomeDB or predicted by DeepC to disrupt local chromatin architecture.

| Putative craniofacial enhancer | Variants | Ref/Alt | rsnumber | Allele Frequency | African Allele Frequency | CADD | RegulomeDB | DeepC (Average score) | Twin set |
| --- | --- | --- | --- | --- | --- | --- | --- | --- | --- |
| chr1:10662943-10683543 | 1:10672212 | C/T | rs578347 | 0.472615 | 0.523557 | 16.78 | 2a | 0.003813 | Twin 1 |
| chr1:156745094-156753838 | 1:156748409 | A/G | rs9426941 | 0.950152 | 0.826689 | 16.03 | 2b | 0.000515 | Twin 1 |
| chr1:46338649-46343089 | 1:46341445 | C/A | rs72885163 | 0.277491 | 0.150814 | 17.02 | 1f | 0.001325 | Twin 2 |
| chr1:234530822-234540909 | 1:234534101 | T/C | rs271762 | 0.494285 | 0.550846 | 17.02 | 1f | 0.00011 | Twin 2 |
| chr2:54454134-54459103 | 2:54457261 | C/T | rs76070960 | 0.1782 | 0.144613 | 16.45 | 1a | 0.000325 | Twin 1 |
| chr2:208264469-208268043 | 2:208266249 | G/C | rs866417196 | 0.011743 | 0.040492 | 15.12 | 2a | 0.002239 | Twin 1 |
| chr2:27427758-27430671 | 2:27428826 | T/C | rs10182937 | 0.399942 | 0.665185 | 17.18 | 1f | 0.00275 | Twin 2 |
| chr2:42128860-42129660 | 2:42129360 | G/T | rs75709763 | 0.017722 | 0.036622 | 15.37 | 1b | 0.000142 | Twin 2 |
| chr2:56183566-56185919 | 2:56184022 | T/C | rs960490 | 0.894069 | 0.759899 | 15.15 | 1f | 0.001309 | Twin 2 |
| chr2:106192661-106195665 | 2:106194271 | A/G | rs7604916 | 0.995611 | 0.932369 | 17.63 | 1f | 0.007447 | Twin 2 |
| chr3:11631986-11645525 | 3:11638311 | C/T | rs13315116 | 0.037271 | 0.083627 | 18.28 | 2a | 9.08E-05 | Twin 2 |
| chr4:95180449-95182049 | 4:95181709 | G/A | rs116469382 | 0.028305 | 0.045222 | 18.81 | 1b | 0.000351 | Twins 1 |
| chr4:165871563-165875863 | 4:165873677 | T/C | rs1460072 | 0.206694 | 0.514649 | 15.37 | 1b | 0.000986 | Twins 1 |
| chr5:68187772-68187972 | 5:68187916 | A/G | rs7734077 | 0.079095 | 0.094314 | 18.42 | 2b | 0.000684 | Twins 1 |
| chr5:88888583-88888783 | 5:88888774 | C/T | rs7737567 | 0.074698 | 0.200542 | 15.56 | 2b | 0.000107 | Twins 1 |
| chr6:35317247-35319093 | 6:35317766 | G/C | rs6937088 | 0.827069 | 0.553429 | 15.55 | 1f | 0.000234 | Twin 1 |
| chr6:149716495-149719449 | 6:149718012 | G/A | rs12174349 | 0.407526 | 0.533938 | 16.22 | 1b | 0.005204 | Twin 1 |
| chr7:16644185-16648299 | 7:16646245 | C/T | rs4145343 | 0.51578 | 0.573303 | 16.91 | 1f | 0.001487 | Twin 2 |
| chr7:138459483-138462080 | 7:138461033 | C/T | rs112619223 | 0.318039 | 0.414527 | 16.1 | 1a | 0.002654 | Twin 2 |
| chr8:73291563-73296161 | 8:73294347 | G/A | rs7819550 | 0.184342 | 0.182794 | 21.2 | 1b | 0.000291 | Twin 2 |
| chr9:112749742-112752238 | 9:112751649 | T/C | rs77365417 | 0.029369 | 0.097558 | 18.79 | 2b | 0.000493 | Twin 1 |
| chr9:134064600-134069601 | 9:134067584 | G/T | rs13287061 | 0.356552 | 0.427342 | 17.29 | 1f | 0.006957 | Twin 1 |
| chr9:19229575-19233135 | 9:19230741 | C/T | rs10481512 | 0.503863 | 0.332835 | 17.3 | 1f | 0.000645 | Twin 2 |
| chr10:87860112-87869716 | 10:87864984 | C/T | rs34370865 | 0.010327 | 0.044353 | 18.41 | 2b | 0.000154 | Twin 1 |
| chr10:103820042-103820642 | 10:103820536 | G/A | rs74490785 | 0.036724 | 0.126921 | 16.06 | 1b | 0.002013 | Twin 1 |
| chr10:27618142-27618971 | 10:27618194 | G/T | rs1168572 | 0.848449 | 0.717012 | 15.01 | 1f | 0 | Twin 2 |
| chr10:70381441-70383664 | 10:70382614 | A/T | rs7921619 | 0.576953 | 0.414548 | 15.44 | 1b | 0.002733 | Twin 2 |
| chr11:62507728-62508928 | 11:62508583 | A/C | rs7123692 | 0.083646 | 0.231639 | 17.71 | 1b | 0.004026 | Twin 1 |
| chr12:76557684-76560552 | 12:76558982 | T/C | rs4761428 | 0.797315 | 0.839249 | 15.96 | 1f | 0.001739 | Twin 1 |
| chr12:120684442-120690756 | 12:120686504 | C/T | rs11065212 | 0.097824 | 0.289526 | 18.77 | 1f | 0.003854 | Twin 1 |
| chr13:106527881-106536620 | 13:106535549 | G/A | rs9558782 | 0.260683 | 0.309271 | 15.1 | 1f | 0.00144 | Twin 2 |
| chr14:59187475-59190890 | 14:59188743 | C/A | rs17833769 | 0.155788 | 0.047974 | 21.4 | 1f | 0.000781 | Twin 2 |
| chr15:22663945-22665989 | 15:22664953 | T/C | rs374154068 | 0.056255 | 0.189233 | 15.78 | 1f | 0.001272 | Twin 1 |
| chr15:22663945-22665989 | 15:22664953 | T/C | rs374154068 | 0.056255 | 0.189233 | 15.78 | 1f | 0.001272 | Twin 2 |
| chr16:49853251-49860331 | 16:49854399 | G/C | rs2241216 | 0.250003 | 0.298572 | 16.53 | 1f | 0.000208 | Twin 1 |
| chr17:7571062-7575050 | 17:7573388 | C/T | rs75853747 | 0.133574 | 0.163188 | 20.2 | 1f | 0.000763 | Twin 1 |
| chr17:18229086-18232086 | 17:18230474 | C/T | rs3889544 | 0.773793 | 0.826844 | 21.2 | 1b | 0.000403 | Twin 1 |
| chr17:83069524-83073924 | 17:83070457 | G/A | rs56260025 | 0.474972 | 0.312172 | 17.1 | 1f | #N/A | Twin 2 |
| chr18:44678099-44685642 | 18:44680383 | C/T | rs117763335 | 0.179228 | 0.069536 | 17.64 | 1f | 0.00025 | Twin 1 |
| chr18:70288088-70291543 | 18:70289064 | G/A | rs41537545 | 0.163337 | 0.170212 | 17.45 | 1f | 0.002633 | Twin 2 |
| chr19:45404370-45407207 | 19:45405792 | C/T | rs1005165 | 0.217578 | 0.278345 | 18.27 | 1b | 0.000139 | Twin 1 |
| chr20:47788656-47790256 | 20:47789190 | A/G | rs7270428 | 0.188621 | 0.2465 | 16.3 | 2b | 0.000704 | Twin 1 |
| chr20:50242135-50245177 | 20:50243220 | G/C | rs6063492 | 0.330945 | 0.72947 | 16.38 | 1a | 0.000491 | Twin 1 |
| chr20:50686155-50688441 | 20:50686966 | A/G | rs4809809 | 0.611217 | 0.452487 | 15.08 | 1f | 0.000108 | Twin 1 |
| chr22:38698586-38703129 | 22:38700597 | T/C | rs6519133 | 0.421219 | 0.79123 | 15.49 | 1f | 0.002003 | Twin 1 |
| chr22:43329154-43329835 | 22:43329829 | A/T | rs57698971 | 0.024039 | 0.069043 | 17.68 | 1f | 9.4E-05 | Twin 2 |
| chr22:43329154-43329835 | 22:43329829 | A/T | rs57698971 | 0.024039 | 0.069043 | 17.68 | 1f | 9.4E-05 | Twin 2 |

**Figure S5:** UCSC Genome Browser (GRCh38) view of the genomic region containing a putative enhancer at chr13:19861036-19865976. The enhancer (vertical blue bar) resides within the same topologically associated domain (TAD) as the *MPHOSPH8* gene. Hi-C contact maps (red triangular matrix) reveal chromatin interactions between the enhancer and the *MPHOSPH8* gene.

Rao et al., Cell. (2014).

Wilderman et al., Cell Rep (2018).

**Figure S6:** UCSC Genome Browser (GRCh38) view of the genomic region harboring a putative enhancer at chr2:55045248-55052196. The enhancer (vertical blue bar) is located within the same topologically associated domain (TAD) as the *SPTBN1* gene, as supported by CNCC chromatin interaction data (horizontal blue bar). Hi-C interaction maps (red triangular matrix) demonstrate physical contacts between the enhancer and the *SPTBN1* gene.

Rao et al., Cell. (2014).

Wilderman et al., Cell Rep (2018).

**Figure S7:** Sequence logos representing binding sites for transcription factors that are predicted to be altered (loss and gain) by the variants in the prioritized putative craniofacial enhancers: ZNF528 (A), KLF16 (B), KLF5 (C), SMAD5 (D) and SBTB14 (E).

Castro-Mondragon JA, et al., *Nucleic Acids Res (*2022).

**Figure S8:** Single-nucleus RNA-seq co-projection of human craniofacial tissues at CS17 showing the expression of *COL27A1* and transcription factors affected by variants within the enhancer at chr9:114286920-114289120. (A) UMAP visualization depicting *COL27A1* expression across distinct craniofacial cell populations. (B) Heatmap illustrating scaled gene expression by cell type for *COL27A1* and transcription factors predicted to gain or lose binding sites due to enhancer-associated variants.

Yankee TN et al., Nat Commun (2023).

**Figure S9:** Single-nucleus RNA-seq projection of human craniofacial tissue illustrating the expression of *MPHOSPH8* and transcription factors impacted by chr13:19861036-19865976 enhancer-associated variant. (A) UMAP representation showing *MPHOSPH8* expression across distinct cell populations. (B) Heatmap displaying scaled gene expression by cell type for *MPHOSPH8* and transcription factors predicted to gain or lose binding sites as a result of variants within the enhancer region chr13:19861036-19865976.

Yankee TN et al., Nat Commun (2023).

**Figure S10**: Heatmap displaying scaled gene expression by Carnegie stage for *MPHOSPH8* and transcription factors predicted to gain or lose binding sites because of variants within the enhancer region chr13:19861036-19865976.

Yankee TN et al., Nat Commun (2023).

**Figure S11:** Single-nucleus RNA-seq projection of human craniofacial tissue highlighting the expression of *SPTBN1* and transcription factors influenced by chr2:55,045,248–55,052,196 enhancer variants. (A) UMAP visualization of *SPTBN1* expression across craniofacial cell populations. (B) Heatmap showing scaled gene expression by cell type for *SPTBN1* and transcription factors predicted to gain or lose binding sites due to variants within the enhancer region chr2:55,045,248–55,052,196.

Yankee TN et al., Nat Commun (2023).

**Table S5:** Discordant deleterious common noncoding variants predicted to be functional or to alter local chromatin architecture and previously reported as genome-wide significant with any trait in the GWAS Catalog

| **Putative craniofacial enhancer** | **Variants** | **Ref/Alt** | **Rs number** | **MAF (Global)** | **MAF (African)** | **CADD score** | **RegulomeDB** | **DeepC (Average score)** | **Nearby CF gene** | **Twin set** |
| --- | --- | --- | --- | --- | --- | --- | --- | --- | --- | --- |
| chr1:10662743-10683743 | 1:10672212 | C/T | rs578347 | 0.472615 | 0.523557 | 16.78 | 2a | 0.003813 | *KIF1B* | Twin 1 |
| chr1:234530822-234540909 | 1:234534101 | T/C | rs271762 | 0.494285 | 0.550846 | 17.02 | 1f | 0.00011 | Nil | Twin 2 |
| chr2:54454134-54459103 | 2:54457261 | C/T | rs76070960 | 0.1782 | 0.144613 | 16.45 | 1a | 0.000325 | *SPTBN1* | Twin 1 |
| chr4:165871563-165875863 | 4:165873677 | T/C | rs1460072 | 0.206694 | 0.514649 | 15.37 | 1b | 0.000986 | *TLL1* | Twins 1 |
| chr6:35317247-35319093 | 6:35317766 | G/C | rs6937088 | 0.827069 | 0.553429 | 15.55 | 1f | 0.000234 | *SCUBE3* | Twin 1 |
| chr7:16644303-16647385 | 7:16646245 | C/T | rs4145343 | 0.51578 | 0.573303 | 16.91 | 1f | 0.001487 | *SOSTDC1* and *ANKMY2* | Twin 2 |
| chr8:73292327-73294954 | 8:73294347 | G/A | rs7819550 | 0.184342 | 0.182794 | 21.2 | 1b | 0.000291 | *TERF1* and *RDH10* | Twin 2 |
| chr9:134065477-134067676 | 9:134067584 | G/T | rs13287061 | 0.356552 | 0.427342 | 17.29 | 1f | 0.006957 | *ADAMTSL2* and *RXRA* | Twin 1 |
| chr18:44678250-44684585 | 18:44680383 | C/T | rs117763335 | 0.179228 | 0.069536 | 17.64 | 1f | 0.00025 | Nil | Twin 1 |
| chr19:45402592-45407260 | 19:45405792 | C/T | rs1005165 | 0.217578 | 0.278345 | 18.27 | 1b | 0.000139 | *BLOC1S3, EXOC3L2* and *OPA3* | Twin 1 |
| chr22:38699308-38702498 | 22:38700597 | T/C | rs6519133 | 0.421219 | 0.79123 | 15.49 | 1f | 0.002003 | Nil | Twin 1 |

**Figure S12**: LacZ reporter activity observed in the neural tube of mouse embryos following random transgenesis driven by the VISTA enhancer element hs289.0 (chr1:10672013–10673061). This enhancer overlaps with our putative craniofacial enhancer region chr1:10662943–10683543.

**Table S6:** Transcription factors predicted by JASPAR to show statistically significant gains or losses of binding in the presence of deleterious common noncoding variants that are predicted to be functional or to alter local chromatin architecture and that have been previously reported as genome-wide significant for at least one trait in the GWAS Catalog.

| **chr1:10672212 C>T** | | | | | | |  |
| --- | --- | --- | --- | --- | --- | --- | --- |
| **TF** | **Class** | **P value (ref)** | **P value (Alt)** | **WT score** | **MT Score** | **Joint (S) Score** | **Implicated in CF development** |
| *EGR3* | C2H2 zinc finger factors | 0.00048 | NA | 0.7403 | 0.7291 | -0.0216 | No |
| *SP3* | C2H2 zinc finger factors | 0.00006 | NA | 0.8946 | 0.8657 | -0.0972 | Yes |
| *SP8* | C2H2 zinc finger factors | 0.00005 | NA | 0.8811 | 0.7897 | -0.2816 | Yes |
| *KLF17* | C2H2 zinc finger factors | 0.00018 | NA | 0.7836 | 0.7692 | -0.0315 | No |
| *SP5* | C2H2 zinc finger factors | 0 | NA | 1 | 0.8542 | -0.7660 | No |
| *KLF11* | C2H2 zinc finger factors | 0.00016 | NA | 0.8508 | 0.8508 | 0 | No |
| **chr1:234534101 T>C** | | | | | | |  |
| **TF** | **Class** | **P value (ref)** | **P value (Alt)** | **WT score** | **MT Score** | **Joint (S) Score** | **Implicated in CF development** |
| *FOXH1* | Fork head/winged helix factors | 0.00016 | NA | 0.8968 | 0.7755 | -0.3917 | Yes |
| *NFIX* | SMAD/NF-1 DNA-binding domain factors | NA | 0.0006 | 0.9325 | 0.9390 | 0.0280 | Yes |
| *MSANTD3* | Tryptophan cluster factors | NA | 0.00025 | 0.7872 | 0.8813 | 0.2897 | No |
| *NFIA* | SMAD/NF-1 DNA-binding domain factors | NA | 0.00138 | 0.8204 | 0.8204 | 0 | Yes |
| **chr2:54457261 C>T** | | | | | | |  |
| **TF** | **Class** | **P value (ref)** | **P value (Alt)** | **WT score** | **MT Score** | **Joint (S) Score** | **Implicated in CF development** |
| *SP9* | C2H2 zinc finger factors | 0.0016 | NA | 0.7804 | 0.7544 | -0.0563 | No |
| *NKX2-4* | Homeo domain factors | 0.0014 | NA | 0.8554 | 0.8030 | -0.1474 | No |
| *TAL1::TCF3* | Basic helix-loop-helix factors (bHLH) | NA | 0.00146 | NA | NA | NA | No |
| **chr4:165873677 T>C** | | | | | | |  |
| **TF** | **Class** | **P value (ref)** | **P value (Alt)** | **WT score** | **MT Score** | **Joint (S) Score** | **Implicated in CF development** |
| *TCF3* | Basic helix-loop-helix factors (bHLH) | NA | 7.44E-05 | 0.8263 | 0.8396 | 0.0354 | No |
| *TFAP2C* | Basic helix-span-helix factors (bHSH) | NA | 0.000451922 | 0.7952 | 0.8430 | 0.1282 | No |
| *ZBTB33* | C2H2 zinc finger factors | NA | 0.000762953 | 0.7122 | 0.7748 | 0.1326 | No |
| *TFAP2B* | Basic helix-span-helix factors (bHSH) | NA | 0.000365 | 0.8582 | 0.8617 | 0.0102 |  |
| *TCF4* | Basic helix-loop-helix factors (bHLH) | NA | 0.00075 | 0.8037 | 0.8162 | 0.0305 | No |
| *GLI3* | C2H2 zinc finger factors | NA | 0.000116 | 0.8188 | 0.8584 | 0.1131 | Yes |
| *TCF12* | Basic helix-loop-helix factors (bHLH) | NA | 8.7E-05 | 0.7842 | 0.8879 | 0.3265 | No |
| **chr6:35317766 G>C** | | | | | | |  |
| **TF** | **Class** | **P value (ref)** | **P value (Alt)** | **WT score** | **MT Score** | **Joint (S) Score** | **Implicated in CF development** |
| *NFIC* | SMAD/NF-1 DNA-binding domain factors | 0.000341892 | NA | 0.9654 | 0.7630 | -0.7788 | Yes |
| *TCF4* | Basic helix-loop-helix factors (bHLH) | 0.000659481 | NA | 0.8128 | 0.6927 | -0.2820 | No |
| *ASCL1* | Basic helix-loop-helix factors (bHLH) | 0.001813889 | NA | 0.7439 | 0.6301 | -0.2180 | Yes |
| *OSR1* | C2H2 zinc finger factors | 0.001621246 | NA | 0.7840 | 0.6811 | -0.2220 | No |
| *HINFP* | C2H2 zinc finger factors | NA | 0.000884891 | 0.6200 | 0.6434 | 0.0355 | No |
| *KLF5* | C2H2 zinc finger factors | NA | 0.002091408 | 0.8359 | 0.8359 | 0 | Yes |
| *ZIC1* | C2H2 zinc finger factors | NA | 0.001045279 | 0.7695 | 0.8354 | 0.1709 | No |
| *GLIS2* | C2H2 zinc finger factors | NA | 0.001350131 | 0.8095 | 0.8405 | 0.0826 | No |
| *ZIC4* | C2H2 zinc finger factors | NA | 0.001080139 | 0.8034 | 0.8495 | 0.1269 | No |
| *ZIC5* | C2H2 zinc finger factors | NA | 0.001366451 | 0.7933 | 0.8321 | 0.1001 | Yes |
| *ZBTB14* | C2H2 zinc finger factors | NA | 0.001165628 | 0.7126 | 0.7866 | 0.1622 | No |
| *SP5* | C2H2 zinc finger factors | NA | 0.000325203 | 0.7261 | 0.8734 | 0.4224 | No |
| **chr7:16646245 C>T** | | | | | | |  |
| **TF** | **Class** | **P value (ref)** | **P value (Alt)** | **WT score** | **MT Score** | **Joint (S) Score** | **Implicated in CF development** |
| *TFAP2A* | Basic helix-span-helix factors (bHSH) | 0.00173831 | NA | 0.9473 | 0.8539 | -0.4003 | Yes |
| *TFAP2B* | Basic helix-span-helix factors (bHSH) | 0.001774311 | NA | 0.8017 | 0.7222 | -0.1827 | No |
| *TFAP2E* | Basic helix-span-helix factors (bHSH) | 0.002415657 | NA | 0.7373 | 0.6941 | -0.0824 | No |
| *ZFP57* | C2H2 zinc finger factors | 0.000372052 | NA | 0.6635 | 0.6450 | -0.0294 | No |
| *KLF2* | C2H2 zinc finger factors | 0.002773285 | NA | 0.7929 | 0.7929 | 0 | Yes |
| *ZNF274* | C2H2 zinc finger factors | NA | 0.00151 | 0.6361 | 0.6678 | 0.0508 | No |
| *SP5* | C2H2 zinc finger factors | NA | 0.002688408 | 0.7261 | 0.8734 | 0.4224 | No |
| **chr8:73294347 G>A** | | | | | | |  |
| **TF** | **Class** | **P value (ref)** | **P value (Alt)** | **WT score** | **MT Score** | **Joint (S) Score** | **Implicated in CF development** |
| *TFAP2C* | Basic helix-span-helix factors (bHSH) | 0.000441272 | NA | 0.7594 | 0.6890 | -0.1423 | No |
| *CTCFL* | C2H2 zinc finger factors | 0.000582576 | NA | 0.8175 | 0.7176 | -0.2403 | No |
| *SNAI3* | C2H2 zinc finger factors | 0.000892639 | NA | 0.8244 | 0.7371 | -0.2161 | No |
| *PATZ1* | C2H2 zinc finger factors | 0.000578225 | NA | 0.8498 | 0.7484 | -0.2738 | No |
| *SPIC* | Tryptophan cluster factors | NA | 0.000607524 | 0.7182 | 0.8158 | 0.2336 | No |
| *ELK3* | Tryptophan cluster factors | NA | 0.001062393 | 0.7309 | 0.7482 | 0.0341 | No |
| **chr9:134067584 G>T** | | | | | | |  |
| **TF** | **Class** | **P value (ref)** | **P value (Alt)** | **WT score** | **MT Score** | **Joint (S) Score** | **Implicated in CF development** |
| *HES2* | Basic helix-loop-helix factors (bHLH) | 0.000430107 | NA | 0.8580 | 0.7225 | -0.3697 | No |
| *TCFL5* | Basic helix-loop-helix factors (bHLH) | 0.000617027 | NA | 0.9341 | 0.8091 | -0.4794 | No |
| *KLF12* | C2H2 zinc finger factors | 0.000816345 | NA | 0.8496 | 0.8351 | -0.0401 | No |
| *HES5* | Basic helix-loop-helix factors (bHLH) | 0.000884056 | NA | 0.6600 | 0.5717 | -0.1382 | No |
| *HES1* | Basic helix-loop-helix factors (bHLH) | 0.000195503 | NA | 0.9227 | 0.7862 | -0.4882 | Yes |
| *TFDP1* | Fork head/winged helix factors | 0.001000166 | NA | 0.8207 | 0.7373 | -0.2041 | No |
| *ZBTB14* | C2H2 zinc finger factors | 0.000306129 | NA | 0.8393 | 0.7316 | -0.2788 | No |
| *SP1* | C2H2 zinc finger factors | 0.000621796 | NA | 0.8238 | 0.8238 | 0 | No |
| *E2F6* | Fork head/winged helix factors | 0.000871629 | NA | 0.8573 | 0.8573 | 0 | Yes |
| *E2F1* | Fork head/winged helix factors | NA | 0.0022223 | 0.7421 | 0.6343 | -0.2058 | No |
| *NR2C2* | Nuclear receptors with C4 zinc fingers | NA | 0.000996487 | 0.7786 | 0.7969 | 0.0418 | No |
| *TFAP2A* | Basic helix-span-helix factors (bHSH) | NA | 0.002168655 | 0.8777 | 0.8807 | 0.0095 | Yes |
| *TFAP2B* | Basic helix-span-helix factors (bHSH) | NA | 0.00284189 | 0.7959 | 0.8187 | 0.0561 | No |
| **chr18:44680383 C>T** | | | | | | |  |
| **TF** | **Class** | **P value (ref)** | **P value (Alt)** | **WT score** | **MT Score** | **Joint (S) Score** | **Implicated in CF development** |
| *TFAP2B* | Basic helix-span-helix factors (bHSH) | 0.001119852 | NA | 0.7746 | 0.7240 | -0.1074 | No |
| *TFAP2C* | Basic helix-span-helix factors (bHSH) | 0.000339855 | NA | 0.7733 | 0.7463 | -0.0572 | No |
| *TFAP2A* | Basic helix-span-helix factors (bHSH) | 0.001181081 | NA | 0.9560 | 0.8360 | -0.5209 | Yes |
| *TFAP2E* | Basic helix-span-helix factors (bHSH) | 0.000338793 | NA | 0.9037 | 0.7881 | -0.3869 | No |
| *ZBTB6* | C2H2 zinc finger factors | 0.000306085 | NA | 0.8402 | 0.7857 | -0.1444 | No |
| *PATZ1* | C2H2 zinc finger factors | 0.000778139 | NA | 0.7936 | 0.7936 | 0 | No |
| *STAT1* | STAT domain factors | NA | 0.000875235 | 0.6564 | 0.6791 | 0.0374 | No |
| *MYOG* | Basic helix-loop-helix factors (bHLH) | NA | 0.000803769 | 0.7047 | 0.8273 | 0.3019 | No |
| *ZBED1* | C2H2 zinc finger factors | NA | 0.000750914 | 0.7232 | 0.8021 | 0.1815 | No |
| *ZBTB7A* | C2H2 zinc finger factors | NA | 0.000767961 | 0.6742 | 0.6257 | -0.0788 | No |
| *ELK3* | Tryptophan cluster factors | NA | 0.000684977 | 0.7477 | 0.7831 | 0.0773 | No |
| *ETV4* | Tryptophan cluster factors | NA | 0.001153946 | 0.7713 | 0.7960 | 0.0563 | No |
| *OSR1* | C2H2 zinc finger factors | NA | 0.001410484 | 0.7169 | 0.8099 | 0.2186 | No |
| *SMAD5* | SMAD/NF-1 DNA-binding domain factors | NA | 0.001409531 | 0.7846 | 0.7912 | 0.0148 | Yes |
| *ZFP57* | C2H2 zinc finger factors | NA | 0.001004398 | 0.7470 | 0.8025 | 0.1286 | No |
| *ASCL1* | Basic helix-loop-helix factors (bHLH) | NA | 0.000982732 | 0.7567 | 0.8419 | 0.2249 | Yes |
| *BHLHE22* | Basic helix-loop-helix factors (bHLH) | NA | 0.001095772 | 0.7010 | 0.7010 | 0 | No |
| *ZNF549* | C2H2 zinc finger factors | NA | 0.00076437 | 0.7045 | 0.8017 | 0.2221 | No |
| **chr19:45405792 C>T** | | | | | | |  |
| **TF** | **Class** | **P value (ref)** | **P value (Alt)** | **WT score** | **MT Score** | **Joint (S) Score** | **Implicated in CF development** |
| *KLF6* | C2H2 zinc finger factors | 0.003073931 | NA | 0.7724 | 0.6434 | -0.2664 | No |
| *MSANTD3* | Tryptophan cluster factors | NA | 0.00297451 | 0.6783 | 0.7884 | 0.2401 | No |
| *NR1D2* | Nuclear receptors with C4 zinc fingers | NA | 0.000161198 | 0.7974 | 0.8427 | 0.1214 | No |
| **chr22:38700597 T>C** | | | | | | |  |
| **TF** | **Class** | **P value (ref)** | **P value (Alt)** | **WT score** | **MT Score** | **Joint (S) Score** | **Implicated in CF development** |
| *SPDEF* | Tryptophan cluster factors | 0.002721071 | NA | 0.7394 | 0.6513 | -0.1677 | No |
| *TBX2* | T-Box factors | 0.003237009 | NA | 0.7880 | 0.7708 | -0.0382 | Yes |
| *KLF10* | C2H2 zinc finger factors | 0.003543854 | NA | 0.7933 | 0.7933 | 0 | No |
| *E2F4* | Fork head/winged helix factors | 0.001017191 | NA | 0.8211 | 0.8470 | 0.0708 | Yes |
| *SP2* | C2H2 zinc finger factors | NA | 0.00170517 | 0.8191 | 0.8254 | 0.0159 | No |
| *KLF14* | C2H2 zinc finger factors | NA | 0.002849579 | 0.7378 | 0.8026 | 0.1499 | No |
| *NR1I3* | Nuclear receptors with C4 zinc fingers | NA | 0.003078461 | 0.6634 | 0.7941 | 0.2878 | No |
| *SMAD5* | SMAD/NF-1 DNA-binding domain factors | NA | 0.002876282 | 0.7558 | 0.7602 | 0.0090 | Yes |
| *ZBTB14* | C2H2 zinc finger factors | NA | 0.000483334 | 0.7425 | 0.8203 | 0.1904 | No |

Figure S13: A proposed overarching genetic mechanism underlying phenotypic discordance in monozygotic twins.
